# Between- and within-person effects of stress on emotional eating in women: a longitudinal study over 49 days

**DOI:** 10.1017/S0033291722002185

**Published:** 2023-08

**Authors:** Natasha Fowler, Megan E. Mikhail, Michael Neale, Pamela K. Keel, Debra K. Katzman, Cheryl L. Sisk, S. Alexandra Burt, Kelly L. Klump

**Affiliations:** 1 Department of Behavioral Neuroscience, Oregon Health & Science University, Portland, Oregon, USA; 2 Department of Psychology, Michigan State University, East Lansing, Michigan, USA; 3 Department of Psychiatry, Human Genetics, and Psychology, Virginia Commonwealth University, Richmond, Virginia, USA; 4 Department of Psychology, Florida State University, Tallahassee, Florida, USA; 5 Division of Adolescent Medicine, Department of Pediatrics, The Hospital for Sick Children, University of Toronto, Toronto, Ontario, Canada; 6 Neuroscience Program, Michigan State University, East Lansing, Michigan, USA

**Keywords:** Stress, binge eating, emotional eating, cortisol, women, longitudinal, daily diary

## Abstract

**Background:**

Stress is associated with binge eating and emotional eating (EE) cross-sectionally. However, few studies have examined stress longitudinally, limiting understanding of how within-person fluctuations in stress influence EE over time and whether stress is a risk factor or consequence of EE. Additionally, little is known regarding how the biological stress response relates to EE.

**Methods:**

We used an intensive, longitudinal design to examine *between-person* and *within-person* effects of major life stress, daily stress, and cortisol on EE in a population-based sample of women (*N* = 477; ages 15–30; *M* = 21.8; s.d. = 3.0) from the Michigan State University Twin Registry. Participants reported past year major life stress, then provided daily ratings of EE and stress for 49 consecutive days. Hair cortisol concentration (HCC) was collected as a longitudinal biological stress measure.

**Results:**

Women reported greater EE when they experienced greater mean stress across days (between-person effects) or greater stress relative to their own average on a given day (within-person effects). Daily stress was more strongly associated with EE than major life stress. However, the impact of daily stress on EE was amplified in women with greater past year major life stress. Finally, participants with lower HCC had increased EE.

**Conclusions:**

Findings confirm longitudinal associations between stress and EE in women, and highlight the importance of *within-person* shifts in stress in EE risk. Results also highlight HCC as a novel biological stress measure that is significantly associated with EE and may overcome limitations of prior physiological stress response indicators.

Binge eating (BE; overconsumption of food with loss of control) is a central feature of several eating disorders (EDs) and affects ~5% of Americans (Hudson, Hiripi, Pope, & Kessler, 2007). BE is strongly influenced by negative emotions (e.g. guilt, sadness; Hawkins & Clement, 1984), and emotional eating (EE) (i.e. overeating in response to negative emotions; Arnow, Kenardy, & Agras, 1995) is strongly associated with BE in both clinical (Masheb & Grilo, 2006; Ricca et al., 2009) and non-clinical (Stice, Presnell, & Spangler, 2002; van Strien, Engels, van Leeuwe, & Snoek, 2005) populations. EE prospectively predicts BE onset (Stice et al., 2002) and is positively associated with BE severity (Ricca et al., 2009). EE is therefore a useful dimensional construct of BE (Haedt-Matt et al., 2014). Notably, the etiology of EE and BE remains poorly understood. Given the significant negative consequences associated with BE/EE, it is important to understand their development to better identify at-risk individuals and tailor prevention and treatment.

Stress has repeatedly been implicated in the etiology of EE/BE (e.g. Degortes et al., 2014; Pike et al., 2006; Rojo, Conesa, Bermudez, & Livianos, 2006; Smyth et al., 2007). Most studies examined *between-person* effects comparing psychological stress levels and physiological stress responses (e.g. cortisol levels) between women with high *versus* low EE/BE. In general, increased frequency and psychological impact of both major life stress (e.g. death of parent/spouse) and acute, daily stress (e.g. heavy traffic) are associated with increased EE/BE (Becker & Grilo, 2011; Diggins, Woods-Giscombe, & Waters, 2015; Loth, van den Berg, Eisenberg, & Neumark-Sztainer, 2008; Woods, Racine, & Klump, 2010). Findings regarding the between-person effects of the physiological stress response are more mixed. Individuals experiencing greater stress show elevated cortisol levels (Godoy, Rossignoli, Delfino-Pereira, Garcia-Cairasco, & de Lima Umeoka, 2018), which are associated with an increased hedonic value (Adam & Epel, 2007) and palatable food consumption (Dallman et al., 2003; Godfrey et al., 2019). However, other studies show decreased (Larsen, van Ramshorst, van Doornen, & Geenen, 2009; Lavagnino et al., 2014) or no significant differences (Coutinho, Moreira, Spagnol, & Appolinario, 2007; Schulz, Laessle, & Hellhammer, 2011) in basal cortisol between women with and without BE. Differences in tissue type may contribute to divergent findings. For example, salivary cortisol is subject to diurnal fluctuations (Parikh et al., 2018) that may introduce error when examining associations with dysregulated eating. Clearly, additional studies are needed to clarify mixed results.

Far fewer studies have examined *within-person* stress-BE associations. Within-person studies examine changes in stress over time to determine *when* and *how* variations in stress influence EE/BE. The few within-person studies suggest stress is a risk factor for, and not correlate of, BE. Increased daily psychological stress precedes bulimia nervosa behaviors (e.g. BE) in women (Goldschmidt et al., 2014), and women perceive daily stressors as more impactful on days when they BE (Smyth et al., 2007; Wolff, Crosby, Roberts, & Wittrock, 2000). Only one study has examined the influence of stress on one day with BE on subsequent days and found that psychological stress is most strongly associated with BE on the same day (Freeman & Gil, 2004). Additionally, only one within-person study has examined associations between cortisol and BE, finding that their relationship may vary by time of day (i.e. stronger in the morning; Carnell et al., 2018). Additional studies of within-person stress are needed to better understand whether changes in stress prospectively predict EE/BE. Understanding these prospective associations is critical for etiological models and determining whether stress reduction may effectively reduce dysregulated eating.

Given the above, the current study aimed to examine the effects of major life stress and daily stress on EE in women using a rigorous, longitudinal study design that spanned 49 consecutive days. We capitalized on the longitudinal design to conduct *between-* and *within-person* analyses to determine *for whom* stress increases EE, distinguish between stress as a predictor *versus* consequence of EE, and elucidate *when* deviations in a woman's stress are most strongly associated with EE (same day or subsequent days). We also sought to clarify conflicting findings regarding associations between physiological stress responses and EE using a novel cortisol measure, hair cortisol concentration (HCC), that can index cortisol over an extended period and is not subject to diurnal fluctuations that may contribute to mixed findings in past research.

## Methods

### Participants

Participants included 477 female twins (15–30 years old; *M* = 21.8; s.d. = 3.0) from the Michigan State University Twin Registry (MSUTR; Burt & Klump, 2019). The MSUTR is a population-based twin registry that recruits twins through birth records (Burt & Klump, 2019). Participants for the current project were recruited from an ongoing study within the MSUTR (i.e. *A Twin Study of Exogenous Hormone Exposure and Binge Eating; EHE-BE*) that examines effects of combined oral contraceptives (COC) on disordered eating. Inclusion criteria included: (1) member of a same-sex female twin pair; (2) age 15–30; (3) at least one co-twin taking COC for ⩾2.5 months (82.7% of participants were using COCs); (4) if not taking COCs, regular menstrual cycles. Exclusion criteria included: (1) pregnancy within the past 12 months or lactation within the past 6 months; and (2) genetic/medical conditions or medications known to directly influence hormones or appetite/weight (see online Supplementary Material for additional details). Participants taking most psychiatric medications (e.g. SSRIs, SNRIs) were eligible. *EHE-BE* spanned the onset of coronavirus disease 2019 (COVID-19), with 143 participants (30.2%) completing the study after the first US case. Though a prior study from our group found increased BE immediately after pandemic onset (Klump et al., in press), there were no significant differences in between- or within-person associations between stress and EE pre- and post-COVID-19 (all *p*s >0.10; data not shown).

While self-report stress measures were administered to all participants, donation of a hair sample was optional for extra compensation. To participate, participants had to have hair longer than 1 inch that was free of chemical treatments (e.g. dying). Because of these inclusion criteria and the inability to collect hair during COVID-19, the sample size for exploratory analyses is smaller (*n* = 234, 49% of the total sample). Compared to previous MSUTR studies (Burt & Klump, 2019), participants in the full sample (96.2% non-Hispanic/Latinx, 89.3% white, 5.0% Black, 1.3% Asian/Pacific Islander, 4.4% multiracial) and the HCC sample (97.4% non-Hispanic/Latinx, 91.1% white, 3.4% Black, 0.4% Asian/Pacific Islander, 5.5% multiracial) had a higher percentage of non-Hispanic/Latinx and white participants. Participants who provided a hair sample were also on average 1 year older and experienced more lifetime major life stress (*p* < 0.001; see online Supplementary Table S1). Participants providing and not providing HCC samples did not differ significantly on other key variables (i.e. EE, stress impact, race/ethnicity; all *p*'s >0.05; see online Supplementary Table S1).

### Procedures

Participants provided behavioral data after 5:00 pm for 49 consecutive days. Participants were followed for 49 days to capture two transitions between active and inactive COC pills for the aims of the parent study. Questionnaires were completed online (99.3%) or via paper scantrons (0.7%). Participants also completed three in-person assessments at the beginning, midpoint (~day 25), and end (~day 49) of the study. Hair samples for HCC were collected during the last study visit. Between visits, staff contacted participants 1×/week to confirm protocol adherence and answer questions. These procedures were effective for minimizing drop-outs (0.5%) and missing data (89% of daily assessments completed), and identifying twins who were no longer eligible (3% due to pregnancy/medication).

### Measures

#### Daily measures

**Emotional Eating (EE)**. EE was assessed using the Dutch Eating Behavior Questionnaire (DEBQ; van Strien, Frihters, Bergers, & Defares, 1986) EE scale modified with permission to refer to that day. Internal consistencies for the EE scale were excellent in previous research (*α* = 0.93; Klump, Keel, Culbert, & Edler, 2008) and the current sample (49-day average *α* = 0.90). The EE scale differentiates between individuals with and without clinically significant BE (Wardle, 1987) and is significantly correlated with established BE measures (*r*'s = 0.55–0.69; Racine, Culbert, Larson, & Klump, 2009; van Strien et al., 1986) and palatable food intake (van Strien, 2000). This scale also shows a robust response to hormonal fluctuations in population-based samples (Klump et al., 2008), which was important for the aims of the parent study examining hormone-dysregulated eating associations.

**Daily Stress**. The Daily Stress Inventory (DSI; Brantley, Waggoner, Jones, & Rappaport, 1987) was used to assess daily stressors. The DSI is a 60-item, self-report questionnaire that asks participants whether they experienced a range of stressors on that day. Participants rated all events as present/absent, then rated the impact of present stressors from 1 (occurred, but was not stressful) to 7 (caused me to panic). The total impact for events on each day was summed to create a daily impact score. We focused on stress impact rather than stress frequency because perceived stress impact may be more closely associated with dysregulated eating (Hay & Williams, 2013; Rojo et al., 2006; Woods et al., 2010; Wolff et al., 2000). Of note, stress frequency and impact were very highly correlated in our data (*r* = 0.93) and previous studies (*r*s >0.90; Brantley, Dietz, McKnight, Jones, & Tulley, 1988; Kanner, Coyne, Schaefer, & Lazarus, 1981), suggesting these constructs may not be fully separable.

The DSI is highly correlated with other self-report stress measures (e.g. the Hassles Scale; Brantley et al., 1987; Kanner et al., 1981) and endocrine stress measures (e.g. urinary cortisol; Brantley et al., 1988), and has excellent internal consistency in past research (*α* >0.80; Brantley et al., 1987) and the current study (average *α* = 0.94).

#### Non-daily measures

**Major Life Stress**. The Social Readjustment Rating Schedule (SRRS; Holmes & Rahe, 1967) was used to assess major life stress. The SRRS is a 43-item questionnaire that asks whether participants experienced major stressful events (e.g. death of a loved one, illness) over the past year. Each item/event is associated with a pre-determined life change unit (LCU) score from 11 to 100 that indicates its severity. LCUs are summed to create a total score across all events. Participants completed the SRRS for the past 12 months (as specified in the original measure), the 49-day study, and over their lifetime.

Scores on the SRRS are highly correlated (*r* = 0.97) with the Schedule of Recent Events, another major life stress measure (Lei & Skinner, 1980). The SRRS has acceptable internal consistency over longer time spans in past research (*α* = 0.72) and the current study (lifetime *α* = 0.80). Internal consistency is somewhat lower over shorter time spans (e.g. in our project, past 12 months *α* = 0.65, during the study *α* = 0.55) because it is relatively unlikely that multiple significant events would occur within these shorter timeframes (see Cleary (1981) for similar findings in prior research).

**Hair Cortisol Concentration (HCC)**. HCC provides a retrospective measure of cortisol levels over an extended time that is not impacted by diurnal fluctuations (Stalder & Kirschbaum, 2012). Because hair grows approximately 1 cm/month (Wennig, 2000), the first 1.5 cm of hair most proximal to the scalp was collected during the final assessment to provide an index of cortisol secretion over the 49-day study. Hair processing was conducted by the Behavioral Immunology and Endocrinology Laboratory at the University of Colorado, Denver following standard procedures (Hoffman, D'Anna-Hernandez, Benitez, Ross, & Laudenslager, 2017; see online Supplementary Material for procedure details). Inter-assay CV was 9.2% for the high hair control and 11.2% for the low hair control. Intra-assay CV was 1.4%.

Prior studies using HCC have found high test-retest reliability (*r*'s 0.68–0.79; Stalder & Kirschbaum, 2012) and positive associations between HCC and 30-day average salivary cortisol levels (*r*'s = 0.61, *p* = 0.01; Short et al., 2016) and major life stress (*β* = 0.21, *p* = 0.04; Karlén, Ludvigsson, Frostell, Theodorsson, & Faresjö, 2011). Cortisol remains stable in hair for up to 6 months (Kirschbaum, Tietze, Skoluda, & Dettenborn, 2009; Noppe et al., 2014).

During the final assessment, participants completed a brief questionnaire about hair care practices (e.g. chemical straightening) that could influence the reliability/validity of HCC.

#### Covariates

Daily ratings of negative affect (NA) were assessed via the NA scale from the Positive and Negative Affect Schedule (Watson, Clark, & Tellegen, 1988). Internal consistency was good (average *α* = 0.84).

Height and weight were measured using a wall-mounted ruler and digital scale during the three in-person visits to calculate BMI. Because prior work has shown minimal changes in weight across a 45-day period (*M* = −0.20 lb change, s.d. = 3.39; Klump et al., 2013), the average BMI across study visits was used. Because sleep is associated with cortisol levels (Nollet, Wisden, & Franks, 2020), hours of sleep/night was included as a covariate in HCC analyses. Hours of sleep were assessed with the question: ‘How many hours of sleep did you get last night?’ Response options included: 0–4, 4–6, 6–7, 7–8, 8–9, 9–10, 10–11, 11–12, 12–13, and more than 13 h.

### Statistical analyses

#### General modeling approach

Daily stress, major life stress, HCC, BMI, NA, and EE were log transformed to account for positive skew. Mixed linear models (MLMs) were used in all analyses to control for the non-independence of the twin data and repeated measures in within-person/daily analyses. Primary analyses of major life stress focused on the last 12 months to maximize variability in scores, but secondary analyses examined major life stress over the 49-day study and across the lifetime (see online Supplementary Tables S2 and S3). To control for multiple comparisons, effects significant at *p* < 0.01 are reported. Pearson correlations for between- and within-person variables are included in online Supplementary Tables S4–S6.

#### Primary analyses

**Between-Person Analyses**. Between-person analyses examined whether women who reported greater daily stress and major life stress also reported greater EE. All daily measures (i.e. EE, daily stress, NA) were averaged across days and standardized prior to analysis. Two-level MLMs were used to test the main and interactive effects of daily and major life stress on EE, with participants nested within families. Income, age, NA, and BMI were included as covariates.

Because HCC is a cumulative cortisol measure, HCC analyses were necessarily between-person. These analyses were identical to other between-person models, except mean hours of sleep were included as an additional covariate. To confirm that HCC-EE associations were unaffected by participants' hair care practices, analyses were repeated in the 220 women (94% of the HCC sample) without potentially confounding factors for HCC.

**Within-Person Analyses.** Within-person analyses examined whether variations in a woman's daily stress relative to her average were associated with fluctuations in same-day and subsequent-day EE. Daily variables were within-person centered (i.e. a participant's daily value was subtracted from her average) and then standardized. Because major life stress is not a daily variable, it was standardized between- rather than within-person.

Three-level MLMs examined how changes in daily stress and covariates were associated with changes in same-day and subsequent-day EE. Observations were nested within participants, and participants were nested within families. MLMs first examined the impact of daily stress on same-day EE. Then, because chronic exposure to major life stress may sensitize women to the effects of daily stressors (Woods et al., 2010), a second series of models examined the 2-way interaction between major life stress and within-person daily stress. A final set of MLMs examined the predictive effects of daily stress from 1 to 2 days ago. Same-day stress was included in time-lagged models to ensure that any predictive effects of time-lagged stress were beyond those of same-day stress.

## Results

### Descriptive statistics

Descriptive statistics for the full sample are presented in Table 1. Participants showed good variability in EE (average score = 0–2.58; possible range = 0–4). Participants also varied considerably on daily stress and major life stress. Mean values and ranges were consistent with previous population-based studies of EE (e.g. Klump et al., 2013), major life stress (e.g. Woods et al., 2010), and daily stress (e.g. Brantley et al., 1987; Wolff et al., 2000; Woods et al., 2010). While there is no standard range for HCC, participants exhibited ample variability in HCC (mean = 10.71 pg/mg, s.d. = 18.86 pg/mg; range = 1.82–191.2 pg/mg) consistent with other population-based adult samples (e.g. Cieszyński, Jendrzejewski, Wiśniewski, Owczarzak, & Sworczak, 2019; O'Brien, Meyer, Tronick, & Moore, 2017).

**Table 1.** Descriptive information for the full sample (*N* = 477) and HCC sample (*N* = 234)

	Full sample		HCC sample		
Variable	Mean (s.d.)	Observed range	Mean (s.d.)	Observed range	Total possible range
*Daily variables −49-Day Avg*					
Avg. emotional eating	0.34 (*0.42*)	0–2.58	0.37 (*0.47*)	0–2.48	0–4
Avg. stress impact	29.53 (*27.37*)	0.92–253.02	15.08 (*3.68*)	10.38–32.13	0–399
Avg. stress frequency	11.08 (*7.53*)	0.67–56.11	11.81 (*7.94*)	0.91–56.11	0–57
Avg. negative affect	15.26 (*3.89*)	10.38–42.37	1.37 (*0.47*)	1–3.48	0–80
*Non-daily variables*					
Major life stress in last 12 months	151.79 (*217.53*)	0–668	130.46 (*107.38*)	0–668	0–2246
HCC (pg/mg)	–	–	10.71 (*18.86*)	1.82–191.20	–
BMI (kg/m^2^)	24.63 (*5.38*)	17.06–58.12	24.63 (*5.15*)	17.06–54.08	–
Ethnicity/Race/Income	Percent (N)		Percent (N)		
Ethnicity					
Hispanic or Latinx	3.8% (18)	–	2.6% (6)	–	–
Non-Hispanic or Latinx	96.2% (460)	–	97.4% (229)	–	–
Race					
White	89.3% (427)	–	91.1% (214)	–	–
Black or African American	5.0% (24)	–	3.4% (8)	–	–
Asian	1.3% (6)	–	0.4% (1)	–	–
More than one race	4.4% (21)	–	5.1% (12)	–	–
Parental income					
Under $20000	2.1% (10)	–	2.1% (5)	–	–
$ 20 000-$ 40 000	3.6% (17)	–	3.1% (7)	–	–
$ 40 000-$ 60 000	10.9% (52)	–	11.1% (25)	–	–
$ 60 000-$ 100 000	27.8% (133)	–	27.1% (61)	–	–
Over $ 100 000	52.3% (250)	–	56.4% (127)	–	–

Avg., average; BMI, body mass index averaged across the three measurements at the beginning, middle, and end of the 49-day collection period; HCC, hair cortisol concentration; stress impact, daily stress impact; stress frequency, daily stress frequency.

*Note*: Variables reported as 49-day averages represent the non-standardized means and standard deviations (s.d.) for each daily variable across the 49-day collection period.

### Between-Person analyses

**Fig. 1.**
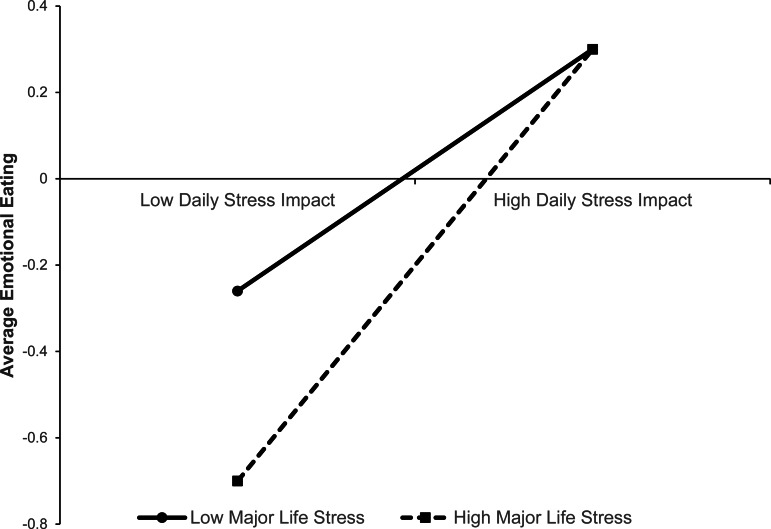
Two-way interaction between average daily stress impact and major life stress in the last 12 months. ‘High’ and ‘low’ values represent 1 s.d. above and below the mean on major life stress and average daily stress impact, respectively.

Controlling for NA, BMI, age, and income, women who experienced higher average daily stress experienced greater mean EE (*β* = 0.35, *p* < 0.001; see Table 2). While major life stress in the last 12 months did not significantly predict average EE (*β* = −0.02, *p* > 0.05), there was a trend-level interaction between average daily stress and major life stress (*β* = 0.11, *p* = 0.03). Specifically, the association between mean daily stress and EE was stronger for women who experienced greater major life stress (see Fig. 1). Finally, after controlling for key confounds, (negative) associations between HCC and average EE were also significant in the full sample of women who provided a hair sample (*β* = −0.14, *p* = 0.007) and the subsample without confounding factors for HCC (*β* = −0.16, *p* = 0.003; Table 3). These findings suggest that women with lower cortisol levels over the 49-day collection period had higher mean EE.

**Table 2.** Results from the between-person MLMs examining main and interactive effects of stress variables and covariates on average levels of emotional eating (*N* = 477)

Variables	*β* (s.d.)	*t* (df)	*p* value
Main effects			
Intercept	−0.05 (*0.04*)	−1.12 (198.64)	0.26
Avg. stress impact	**0.35 (*0.06*)**	**5.77 (148.63)**	**<0.001**
Avg. negative affect	**0.28 (*0.06*)**	**4.35 (218.29)**	**<0.001**
Avg. BMI	0.02 (*0.04*)	0.55 (313.12)	0.59
Age	<0.01 (*0.05*)	0.08 (209.57)	0.94
Income	0.01 (*0.04*)	0.28 (62.67)	0.78
			
Intercept	−0.03 (*0.05*)	−0.72 (198.36)	0.47
Major life stress in the last 12 months	−0.02 (*0.05*)	−0.33 (103.59)	0.75
Avg. negative affect	**0.52 (*0.06*)**	**9.40 (170.10)**	**<0.001**
Avg. BMI	0.06 (*0.05*)	1.24 (283.52)	0.22
Age	−0.01 (*0.05*)	−0.22 (208.42)	0.83
Income	0.01 (*0.05*)	0.17 (57.95)	0.87
Interactions			
Intercept	−0.10 (*0.04*)	−2.20 (206.551)	0.03
Avg. stress impact	**0.39 (*0.06*)**	**6.13 (153.75)**	**<0.001**
Major life stress in the last 12 months	−0.11 (*0.05*)	−2.22 (79.57)	0.03
Avg. stress impact × major life stress in the last 12 months	0.11 (*0.05*)	2.18 (88.64)	0.03
Avg. negative affect	**0.27 (*0.06*)**	**4.14 (202.83)**	**<0.001**
Avg. BMI	0.03 (*0.04*)	0.73 (286.46)	0.47
Age	<0.01 (0.05)	−0.01 (205.04)	0.99
Income	0.01 (*0.04*)	0.15 (55.31)	0.88

MLM, multilevel model; avg., average; BMI, body mass index; stress impact, daily stress impact.

*Note:* All daily variables were averaged across the 49 days of data collection, and BMI was averaged across the 3 study assessments. Betas represent standardized effects. Effects significant at *p* < 0.01 are bolded.

**Table 3.** Results from the between-person, exploratory MLMs examining the effects of hair cortisol concentration (HCC) and covariates on average levels of emotional eating

	*β* (s.d.)	*t* (df)	*p* value
Full HCC sample (*N* = 234)			
Intercept	−0.02 (*0.06*)	−0.35 (126.16)	0.73
HCC	**−0.14 (*0.05*)**	**−2.70 (203.34)**	**0.007**
Avg. negative affect	**0.59 (*0.07*)**	**8.81 (93.28)**	**<0.001**
Avg. BMI	0.03 (*0.05*)	0.48 (179.02)	0.64
Age	0.03 (*0.07*)	0.37 (135.70)	0.71
Avg. hours of sleep/night	0.01 (*0.05*)	0.10 (204.55)	0.92
Income	0.03 (*0.06*)	0.47 (128.11)	0.64
Subsample of women without confounding factors for HCC (*N* = 220)			
Intercept	−0.02 (*0.06*)	−0.38 (120.50)	0.70
HCC	**−0.16 (*0.05*)**	**−2.97(179.83)**	**0.003**
Avg. negative affect	**0.59 (*0.07*)**	**8.49 (81.79)**	**<0.001**
Avg. BMI	0.01 (*0.06*)	0.18 (28.75)	0.86
Age	<0.01 (*0.07*)	0.11 (128.72)	0.91
Avg. hours of sleep/night	<0.01 (*0.05*)	0.07 (177.99)	0.95
Income	0.03 (*0.06*)	0.51 (119.39)	0.61

MLM, multilevel model; avg., average; BMI, body mass index; HCC, hair cortisol concentration.

*Note:* All daily variables were averaged across the 49 days of data collection, and BMI was averaged across the 3 study assessments. Betas represent standardized effects. Effects significant at *p* < 0.01 are bolded.

### Within-person analyses

Increases in same-day stress relative to a person's mean predicted increased daily EE (*β* = 0.15, *p* < 0.001; see Table 4). Daily stress also significantly predicted EE one and two days later, though these effects were weaker (*β* = 0.03–0.04, *p*s <0.001; see Table 4). Counter to hypotheses, interactions between major life stress and within-person changes in daily stress were non-significant (*β*'s ≤0.01, all *p*'s >0.05; see Table 4).

**Table 4.** Results from the within-person MLMs examining main and interactive effects of the same-day and time-lagged stress variables and covariates on daily levels of emotional eating (*N* = 477)

Variables	*β* (s.d.)	*t* (df)	*p* value
Same-day daily stress impact			
Main effects			
Intercept	0.01 (*0.01*)	0.68 (2804.13)	0.50
Same-day stress impact	**0.15 (*0.01*)**	**10.45 (329.19)**	**<0.001**
Negative affect	**0.13 (*0.01*)**	**9.09 (415.05)**	**<0.001**
BMI	<0.01 (*0.03*)	0.06 (112.56)	0.96
Interactions			
	*β* (s.d.)	*t* (df)	*p* value
Intercept	0.01 (*0.01*)	0.81 (2881.49)	0.42
Same-day stress impact	**0.15 (*0.02*)**	**9.95 (327.49)**	**<0.001**
Major life stress in the last 12 months	<0.01 (*0.01*)	0.18 (2933.70)	0.86
Same-day stress impact × major life stress in the last 12 months	<0.01 (*0.01*)	−0.18 (249.34)	0.86
Negative affect	**0.13 (*0.02*)**	**8.82 (406.50)**	**<0.001**
BMI	0.01 (*0.01*)	0.79 (4636.52)	0.43
Daily stress from one day ago (Lagged effects)			
Main effects			
Intercept	−0.02 (*0.01*)	−2.72 (3180.25)	0.01
Same-day stress impact	**0.13 (*0.02*)**	**8.60 (370.53)**	**<0.001**
Stress impact from one day ago	**0.04 (*0.01*)**	**2.68 (344.12)**	**0.008**
Negative affect	**0.13 (*0.01*)**	**9.04 (422.47)**	**<0.001**
BMI	<0.01 (*0.01*)	0.54 (4664.91)	0.59
Interactions			
Intercept	−0.02 (*0.01*)	−2.62 (3088.08)	0.01
Same-day stress impact	**0.13 (*0.02*)**	**8.33 (369.94)**	**<0.001**
Stress impact from one day ago	**0.04 (*0.01*)**	**2.77 (340.22)**	**0.006**
Major life stress in the last 12 months	<0.01 (*0.01*)	0.17 (3179.58)	0.86
Same-day stress impact × major life stress in the last 12 months	0.01 (*0.02*)	0.76 (276.65)	0.45
Stress impact from one day ago × major life stress in the last 12 months	<0.01 (*0.01*)	−0.33 (274.43)	0.74
Negative affect	**0.13 (*0.01*)**	**8.78 (416.59)**	**<0.001**
BMI	<0.01 (*0.01*)	0.51 (4519.89)	0.61
Daily stress from two days ago (Lagged effects)			
Main effects			
Intercept	**−0.03 (*0.01*)**	**−3.49 (3046.27)**	**<0.001**
Same-day stress impact	**0.12 (*0.01*)**	**8.24 (366.51)**	**<0.001**
Stress impact from two days ago	**0.03 (*0.01*)**	**3.00 (318.08)**	**<0.001**
Negative affect	**0.14 (*0.02*)**	**8.83 (425.05)**	**<0.001**
BMI	0.01 (*0.01*)	0.78 (4372.88)	0.43
Interactions			
Intercept	**−0.03 (*0.01*)**	**−3.40 (2946.43)**	**0.001**
Same-day stress impact	**0.12 (*0.02*)**	**8.12 (370.16)**	**<0.001**
Stress impact from two days ago	0.03 (*0.01*)	2.42 (323.26)	0.02
Major life stress in the last 12 months	<0.01 (*0.01*)	0.27 (3038.50)	0.79
Same-day stress impact × major life stress in the last 12 months	0.01 (*0.01*)	0.63 (272.52)	0.53
Stress impact from two days ago × major life stress in the last 12 months	<0.01 (*0.01*)	-0.17 (243.41)	0.86
Negative affect	**0.13 (*0.02*)**	**8.51 (417.74)**	**<0.001**
BMI	0.01 (*0.01*)	0.74 (4223.49)	0.46

MLM, multilevel model; BMI, body mass index; stress impact, daily stress impact.

*Note*: Betas represent standardized effects. Effects significant at *p* < 0.01 are bolded.

### Post-hoc analyses

Non-significant within-person interactions between major life stress and daily stress were somewhat surprising, and raised the question of whether effects might differ depending on the presence of clinically significant eating pathology. We therefore conducted exploratory, post-hoc analyses examining the impact of having lifetime DSM-5 anorexia nervosa, bulimia nervosa, or binge-eating disorder (*n* = 32; 7.6% of participants with complete diagnostic data) or lifetime BE regardless of the presence of a threshold ED (*n* = 45; 10% of participants) on results. ED diagnoses and BE were assessed with the Structured Clinical Interview for DSM (First, Spitzer, Gibbon, & Williams, 1996). Between-person stress-EE associations were unchanged when including history of an ED or BE in the model, though participants with a lifetime ED or BE reported greater EE overall (see online Supplementary Tables S8–S9). When examining within-person effects of stress in the subsamples of participants with a lifetime ED/BE, main effects of major life and daily stress were similar to effects in the full sample, while effect sizes for interactions between major life stress and daily stress tended to be larger (see online Supplementary Tables S10 and S11). Although *p* values were largely non-significant, this was likely due to the much smaller samples.

## Discussion

This is the first longitudinal study to examine *between-* and *within-person* effects of daily stress, major life stress, and HCC on EE in women. Findings indicate that daily stress is a more robust predictor of EE in women than major life stress. Specifically, women reported greater EE when they experienced higher daily stress relative to other women (between-person effects) and their own mean (within-person effects). While shifts in daily stress more strongly predicted same-day EE as compared to subsequent-day EE, prospective associations between stress and EE were evident across at least two days. Lastly, women with lower HCC reported greater EE. Overall, the current study extends prior findings of stress and EE in women by distinguishing the relative influence of daily stress, major life stress, and cortisol on EE, identifying *for whom* stress increases EE, and *when the* effects of stress are the strongest.

Stronger predictive effects of daily stress compared to major life stress on EE may seem surprising given the more severe nature of major life stressors. One possibility is that major life stress may be more important for initiating than for maintaining ongoing EE/BE. Women are 6× more likely to develop disordered eating if they experience chronically high major life stress (Pike et al., 2006), and report elevated major life stress in the year preceding ED onset (Rojo et al., 2006). Past studies have shown that chronic and/or severe stressors, such as trauma (e.g. violence, abuse), are particularly influential in predicting EE/BE (Backholm, Isomaa, & Birgegård, 2013; Palmisano, Innamorati, & Vanderlinden, 2016; Smyth, Heron, Wonderlich, Crosby, & Thompson, 2008; Zelkowtiz, Zerubavel, Zucker, & Copeland, 2021). Because we did not assess trauma specifically, we were unable to examine whether the trauma was more closely associated with EE than other major life stressors. However, this is an important avenue for future research given evidence for the role of trauma specifically in EE/BE.

While major life stress was not directly associated with EE, interaction analyses suggested that daily stress may be more strongly related to EE in women with more major life stress, perhaps especially for women with lifetime BE/EDs (see online Supplementary Tables S10–S11). This is an important finding that may help to better identify women at risk for EE. The observed interaction effect is consistent with past research (Woods et al., 2010), and could indicate that major life stress ‘primes’ perpetuation of EE in women by increasing the impact of more minor stressors in the future. Replication studies are needed to verify results and further elucidate the relationship between major life stress and daily stress on EE across the spectrum of eating pathology.

Same-day stress more strongly predicted daily EE than did prior-day stress. This finding is consistent with the few other daily studies that examined the lagged impact of stress on disordered eating (Barker, Williams, & Galambos, 2006; Freeman & Gil, 2004; Smith et al., 2021). Increased NA may partially mediate these associations (Goldschmidt et al., 2014; Srivastava, Lampe, Michael, Manasse, & Juarascio, 2021), and same-day stress may impact EE more strongly because it triggers an immediate increase in NA (Klatzkin et al., 2019; Steinsbekk, Barker, Llewellyn, Fildes, & Wichstrøm, 2018). Alternatively, results may reflect the difficulty in statistically differentiating the impact of prior-day from same-day stress. Notably, however, correlations between same-day and time-lagged stress were relatively low (*r*'s = 0.10–0.13), suggesting the stressors experienced on each day may be distinct. Additional research is needed to determine whether stronger associations between same-day stress and EE are due to NA, statistical artifacts, or other factors (e.g. greater impact of same-day stress on physiological stress systems).

A novel finding was that lower HCC predicted greater EE, potentially indicating hypoactive hypothalamic–pituitary–adrenal (HPA) axis functioning in women with greater EE. This finding is consistent with some prior research showing a blunted/hypoactive cortisol response to stress in women with EE (Het et al., 2015, 2020; Tomiyama, Dallman, & Epel, 2011; van Strien, Roelofs, & de Weerth, 2013). While overall associations between cortisol and EE/BE are mixed in the literature, this inconsistency may be due in part to diurnal fluctuations in salivary and urinary cortisol that introduce measurement error (Carnell et al., 2018). Because HCC provides a cumulative, longitudinal measure of cortisol that is unaffected by diurnal fluctuations, it may be a particularly useful index of cortisol-EE/BE associations. Interestingly, while hypoactive HPA-axis functioning can develop following chronic stress (Lo Sauro, Ravaldi, Cabras, Faravelli, & Ricca, 2008), we found no significant association between HCC and self-reported daily stress impact assessed concurrently (*r* = 0.02; *p* > 0.05; see online Supplementary Table S6). Other studies have reported a similar lack of association between HCC and self-reported stress (e.g. Braig et al., 2016; O'Brien, Tronick, & Moore, 2013; Schlotz et al., 2008; Streit et al., 2016), indicating that self-report and physiological stress measures such as HCC may be tapping partially distinct aspects of stress. Future studies are needed to better understand the relationship between self-reported and physiological stress measures, and replicate associations between HCC and EE.

Moving forward, it will be important to identify the mechanisms underlying daily stress-EE associations. Acute stress increases activity in mesocorticolimbic regions involved in reward processing (e.g. anterior cingulate cortex, nucleus accumbens) while simultaneously reducing activity in brain regions associated with inhibitory control (e.g. prefrontal cortex) that play an important role in regulating emotions (Dixon, Thiruchselvam, Todd, & Christoff, 2017) and ‘braking’ reward-related behavior such as palatable food consumption (Arnsten, 2015). With decreased regulatory control, it may be more difficult to abstain from dysregulated eating when stress is high. This may be particularly true because stressful events are often accompanied by high NA. High NA concurrent with neurobiological reactions to stress that reduce the capacity for emotion regulation may create a ‘perfect storm’ for EE/BE. Additional work in human and animal models is needed to test this hypothesized mechanism. Research is also needed on other potential psychological mediators and moderators of stress-EE associations, including emotion regulation and personality variables that may impact the likelihood of stress triggering EE.

Before concluding, it is important to note the study limitations. First, while this study sought to examine how stress predicts EE, it is possible that stress and EE exhibit a reciprocal relationship. Despite studies reporting decreases in stress immediately after BE (Smyth et al., 2009), BE is often accompanied by guilt and increases in overall NA (Mikhail, 2021) that may subsequently increase perceived stress. Post-hoc MLMs were conducted to examine whether stress increased following EE (see online Supplementary Table S7). Indeed, EE from one day ago predicted subsequent daily stress (*β* = 0.03; *p* < 0.001), suggesting there may be a reciprocal relationship between EE and stress that can persist over multiple days.

Second, because HCC provided a cumulative measure of cortisol over 49 days, we were only able to examine associations between EE and cortisol at a between-person level. To more fully understand how cortisol influences EE, within-person studies that examine how daily variations in cortisol contribute to daily shifts in EE are needed. Given individual differences in cortisol reactivity to stress (Raspopow, Abizaid, Matheson, & Anisman, 2010; Tomiyama et al., 2011; van Strien et al., 2013) and diurnal variation in cortisol-disordered eating associations (Carnell et al., 2018), assessing within-person effects of cortisol on EE may require multiple measures of cortisol throughout the day.

Lastly, the current sample was young, predominantly non-Hispanic/Latinx, white, and socioeconomically advantaged, and met inclusion criteria that may limit generalizability. Prior studies have reported that women of color experience significantly higher stress than white women due to increased discrimination, oppression, and lower socioeconomic status, among other stressors (Hatch & Dohrenwend, 2007; O'Brien et al., 2017). Therefore, it will be important to replicate this study in a more racially/ethnically and socioeconomically diverse sample. Associations between stress and EE may also change with age. In our sample, age was negatively correlated with NA (*r* = −0.24, *p* < 0.001), stress (*r* = −0.13, *p* = 0.005), and, to a lesser extent, EE (*r* = −0.10, *p* = 0.023). As people age, they may develop better strategies for managing stress, leading to lower average NA and perceived stress that may contribute to lower EE. However, older women may also be more likely to experience stressors such as caregiving demands for children or aging parents, career pressures, or health concerns. Future research should examine how different kinds of stressors may contribute to EE across the lifespan. Additional research is also needed in larger samples of participants with BE and ED diagnoses. Finally, our sample only included women, and research is needed on the relationship between stress and EE in men.

Despite these limitations, the current results have important clinical implications. Clinicians should work with clients with dysregulated eating to decrease stress when possible and develop strategies for managing stressors that may be unavoidable (e.g. problem solving, engaging in self-care and effective emotion regulation). Clinicians should be particularly attentive to the impact of daily stressors on clients with histories of major life stress (e.g. abuse, loss) who may be particularly vulnerable to EE when new stressors arise.

## Supplementary material

For supplementary material accompanying this paper visit https://doi.org/10.1017/S0033291722002185. click here to view supplementary material
