# Supplementary material for: Between- and within-person effects of stress on emotional eating in women: a longitudinal study over 49 days
Source: Psychol Med. 2022 Aug 1;53(11):5167–76. doi: 10.1017/S0033291722002185 (PMC10471857; doi:10.1017/S0033291722002185)
Supplement: Supplementary file 1 [file S0033291722002185sup001.docx]

**Supplemental Material**

**Additional Details Regarding Study Inclusion/Exclusion Criteria**

Medical conditions leading to exclusion included thyroid disorders (Hashimoto Disease, hypothyroidism, hyperthyroidism), polycystic ovarian syndrome (PCOS), endometriosis, diabetes, Turner’s syndrome, and severe developmental disabilities that would significantly interfere with participants’ ability to understand study procedures. Medications leading to exclusion included any medication known to impact the hypothalamic–pituitary–gonadal (HPG) axis, including Spironolactone, Klonopin, Accutane, Rifamycin, steroid medications (e.g., Prednisone), Lithium, and antipsychotic/anticonvulsant medications. Participants were eligible if taking non-steroidal allergy medications (e.g., antihistamines), non-steroidal pain medications (e.g., ibuprofen, trazadone), medications for gastroesophageal reflux (e.g., pantoprazole), or most SSRIs, SNRIs, CNS stimulants, and benzodiazepines. If there was uncertainty regarding whether a medical condition or medication had a direct impact on hormone functioning or appetite/weight, study personnel examined the extant research on that condition or medication to make a determination.

**Procedures for Hair Cortisol Concentration Analysis**

Following standard procedures (Wright et al., 2018), hair samples were obtained by cutting hair from the posterior vertex of the scalp, as close to the scalp as possible. Hair samples were wrapped in aluminum foil for protection and stored at room temperature, as previously described (Wennig, 2000), until they were shipped to the Behavioral Immunology and Endocrinology Laboratory at the University of Colorado, Denver at the Anschutz Medical Campus for analysis. Following the procedures outlined in Hoffman et al. (2017), upon arrival at the lab, hair was ground and cortisol levels were measured using a commercial high sensitivity EIA kit (Salimetrics, LLC, State College, PA) that was conducted according to manufacturer’s instructions as previously described by D’Anna-Hernandez et al. (2011). To calculate inter-assay coefficients of variation (CV), a pooled control of previously ground hair was extracted using the same procedures outlined above and included on each EIA plate in duplicate. Inter-assay CV for the control hair pool was 9.2% for the high hair control and 11.2% for the low hair control and intra-assay CV was 1.4%.

A growing number of studies have used HCC as a measure of cumulative cortisol secretion (see reviews Sander et al., 2020; Stalder et al., 2017; Stalder & Kirschbaum, 2012) and found high test-retest associations between repeated HCC assessments (*r*’s between 0.68-0.79; Stalder & Kirschbaum, 2012), positive associations between HCC and 30-day average salivary cortisol levels (*r*’s = 0.61*,* *p* = .01; Short et al., 2016) and levels of major life stress (β = 0.21, *p* = .04 for stressors such as death of a close relative, serious illness, divorce; Karlén et al., 2011) assessed over the same time period. These data support the validity of HCC as a measure of cumulative cortisol concentration over the study.

**Table S1.** *Comparing descriptive information between participants who did and did not provide a hair sample*.

|  | **HCC sample**  **(*N = 234*)** | **Non-HCC sample**  **(*N = 243*)** | **t-test results** | ***p-value*** |
| --- | --- | --- | --- | --- |
| **Variable** | **Mean (*SD*)** | **Mean (*SD*)** | **t(*df*) = F** |  |
| **Daily Variables –**  **49-Day Avg** |  |  |  |  |
| Avg. emotional eating | 0.37 (*0.47*) | 0.31 (*0.36*) | -1.60(*475*) = 8.00 | .11 |
| Avg. stress impact | 30.67 (*27.01*) | 28.42 (*27.71*) | -0.90(*475*) = 0.16 | .37 |
| Avg. stress frequency | 11.81 (*7.94*) | 10.39 (*7.06*) | -2.07(*475*) = 2.50 | .04 |
| Avg. negative affect | 15.08 (*3.68*) | 15.43 (*4.08*) | 0.99(*475*) = 0.13 | .33 |
|  |  |  |  |  |
| **Non-Daily variables** |  |  |  |  |
| Major life stress in the last  12 months | 130.46 (*107.38*) | 142.69 (*108.88*) | 1.22(*460*) = 0.29 | .23 |
| Major life stress over 49  days | 62.63 (*70.08*) | 71.92 (*73.19*) | 1.39(*460*) = 0.99 | .16 |
| Major life stress across the  lifetime | 109.44 (*168.48*) | 194.51 (*250.94*) | 4.28(*460*) = 27.94 | **<.001** |
| BMI (kg/m^2^) | 24.63 (*5.15*) | 24.63 (*5.70*) | 0.00(*399*) = 0.56 | 1.00 |
|  |  |  |  |  |
| **Ethnicity/Race/Income** | **Percent (N)** | **Percent (N)** | **Z-test of proportions** |  |
| Ethnicity |  |  |  |  |
| Hispanic or Latinx | 2.6% (6) | 4.9% (12) | -0.24 | .81 |
| Non-Hispanic or Latinx | 97.4% (229) | 95.1% (231) | 1.35 | .18 |
| Race (%, *n*) |  |  |  |  |
| White | 91.1% (214) | 87.7% (213) | 1.13 | .26 |
| Black or African American | 3.4% (8) | 6.6% (16) | -0.34 | .73 |
| Asian | 0.4% (1) | 2.1% (5) | -0.14 | .89 |
| More than one race | 5.1% (12) | 3.7% (9) | 0.16 | .87 |
| Income |  |  |  |  |
| Under $20,000 | 2.1% (5) | 2.1% (5) | 0 | 1.00 |
| $20,000-$40,000 | 3.1% (7) | 4.1% (10) | -0.11 | .91 |
| $40,000-$60,000 | 11.1% (25) | 11.1% (27) | 0 | 1.00 |
| $60,000-$100,000 | 27.1% (61) | 30.4% (72) | -0.37 | .71 |
| Over $100,000 | 56.4% (127) | 51.9% (123) | 0.71 | .48 |

*Note:* Avg. = average; BMI = body mass index averaged across the three measurements at the beginning, middle, and end of the 49-day collection period; HCC = hair cortisol concentration. Variables reported as 49-day averages represent the non-standardized means and standard deviations (SD) for each daily variable across the 49-day collection period. Values significant at *p* <.01 are bolded.

**Table S2.** *Results from between-person MLMs examining interactive effects of daily stress with major life stress over the 49-day study period and across the lifetime (N = 477).*

| **Main Effects** | | | |
| --- | --- | --- | --- |
| **Predictors** | **β (*SD*)** | **t (df)** | ***p-value*** |
| Intercept | -0.03 (*0.04*) | -0.74 (196.43) | .46 |
| Major life stress over 49 days | -0.05 (*0.04*) | -1.29 (351.50) | .20 |
| Avg. negative affect | **0.53 (*0.05*)** | **9.75 (163.24)** | **<.001** |
| Avg. BMI | 0.05 (*0.04*) | 1.14 (288.41) | .26 |
| Age | <0.01 (*0.05*) | -0.19 (206.44) | .85 |
| Income | 0.01 (*0.05*) | 0.26 (57.07) | .80 |
| Intercept | -0.03 (*0.04*) | -0.77 (199.27) | .44 |
| Major life stress across the lifetime | -0.06 (*0.04*) | -1.32 (357.70) | .19 |
| Avg. negative affect | **0.53 (*0.05*)** | **9.74 (160.41)** | **<.001** |
| Avg. BMI | 0.05 (*0.04*) | 1.23 (290.52) | .22 |
| Age | -0.01 (*0.05*) | -0.21 (208.84) | .84 |
| Income | 0.01 (*0.05*) | 0.28 (59.40) | .78 |
| **Interaction Effects** | | | |
| **Predictors** | **β (*SD*)** | **t (df)** | ***p-value*** |
| Intercept | -0.02 (*0.04*) | -0.44 (201.06) | .66 |
| Avg. stress impact | **0.36 (*0.06*)** | **6.17 (346.42)** | **<.001** |
| Major life stress over 49 days | -0.08 (*0.04*) | -1.82 (365.03) | .07 |
| Avg. stress impact X major life stress over 49 days | 0.08 (*0.04*) | 1.98 (345.15) | .05 |
| Avg. negative affect | **0.33 (*0.06*)** | **5.59 (345.13)** | **<.001** |
| Avg. BMI | 0.06 (*0.05*) | 1.19 (67.95) | .24 |
| Age | <0.01 (*0.05*) | -0.18 (205.34) | .86 |
| Income | 0.03 (*0.05*) | 0.68 (80.22) | .50 |
| Intercept | -0.07 (*0.04*) | -1.56 (203.58) | .12 |
| Avg. stress impact | **0.35 (*0.06*)** | **5.60 (154.50)** | **<.001** |
| Major life stress across the lifetime | -0.08 (*0.04*) | -2.07 (345.29) | .04 |
| Avg. stress impact X major life stress across the lifetime | -0.04 (*0.05*) | -0.91 (196.32) | .37 |
| Avg. negative affect | **0.27 (*0.07*)** | **4.11 (211.79)** | **<.001** |
| Avg. BMI | 0.03 (*0.04*) | 0.70 (294.34) | .48 |
| Age | <0.01 (*0.05*) | -0.16 (208.03) | .87 |
| Income | <0.01 (*0.04*) | 0.03 (202.41) | .98 |

*Note*: MLM = multilevel model; avg. = average; BMI = body mass index; stress impact = daily stress impact. All daily variables were averaged across the 49 days of the study. Values significant at *p* <.01 are bolded.

**Table S3.** *Results from within-person MLMs examining interactive effects of same-day and time-lagged daily stress with major life stress over the 49-day study period and across the lifetime (N = 477).*

| **Same-Day Daily Stress Impact** | | | |
| --- | --- | --- | --- |
| **Interaction Effects** | | | |
| **Predictors** | **β (*SD*)** | **t (df)** | ***p-value*** |
| Intercept | 0.01 (*0.01*) | 0.80 (2879.56) | .43 |
| Same-day stress impact | **0.15 (*0.01*)** | **9.98 (320.06)** | **<.001** |
| Major life stress over 49 days | <0.01 (*<0.01*) | -0.04 (2870.98) | .97 |
| Same-day stress impact X major life stress  over 49 days | <0.01 (*0.01*) | -0.29 (320.64) | .77 |
| Negative affect | **0.13 (*0.02*)** | **8.83 (406.46)** | **<.001** |
| BMI | 0.01 (*0.01*) | 0.79 (4637.11) | .43 |
| Intercept | 0.01 (*0.01*) | 0.83 (2872.77) | .41 |
| Same-day stress impact | **0.15 (*0.01*)** | **9.97 (315.94)** | **<.001** |
| Major life stress across the lifetime | <0.01 (*0.01*) | 0.38 (2884.66) | .70 |
| Same-day stress impact X major life stress  across the lifetime | <0.01 (*0.01*) | 0.27 (305.11) | .79 |
| Negative affect | **0.13 (*0.02*)** | **8.83 (406.61)** | **<.001** |
| BMI | 0.01 (*0.01*) | 0.79 (4638.27) | .43 |
| **Daily Stress Impact from One Day Ago** | | | |
| **Interaction Effects** | | | |
| **Predictors** | **β (*SD*)** | **t (df)** | ***p-value*** |
| Intercept | -0.02 (*0.01*) | -2.62 (3083.42) | .01 |
| Same-day stress impact | **0.13 (*0.02*)** | **8.39 (361.99)** | **<.001** |
| Stress impact from one day ago | **0.04 (*0.01*)** | **2.82 (335.40)** | **.005** |
| Major life stress over 49 days | <0.01 (*0.01*) | 0.29 (3075.63) | .78 |
| Same-day stress impact X major life stress  over 49 days | 0.01 (*0.02*) | 0.41 (346.57) | .68 |
| Stress impact from one day ago X major  life stress over 49 days | -0.02 (*0.01*) | -1.50 (357.20) | .14 |
| Negative affect | **0.13 (*0.01*)** | **8.78 (416.60)** | **<.001** |
| BMI | <0.01 (*0.01*) | 0.51 (4521.33) | .61 |
| Intercept | -0.02 (*0.01*) | -2.63 (3073.31) | .01 |
| Same-day stress impact | **0.13 (*0.02*)** | **8.43 (356.87)** | **<.001** |
| Stress impact from one day ago | **0.04 (*0.01*)** | **2.78 (332.70)** | **.006** |
| Major life stress across the lifetime | <0.01 (*0.01*) | 0.02 (3097.52) | .99 |
| Same-day stress impact X major life stress  across the lifetime | <0.01 (*0.02*) | 0.16 (342.98) | .87 |
| Stress impact from one day ago X major  life stress across the lifetime | 0.01 (*0.01*) | 0.70 (338.34) | .48 |
| Negative affect | 0.13 (*0.01*) | 8.77 (342.98) | .87 |
| BMI | <0.01 (*0.01*) | 0.51 (4521.98) | .61 |
| **Daily Stress Impact from Two Days Ago** | | | |
| **Interaction Effects** | | | |
| **Predictors** | **β (*SD*)** | **t (df)** | ***p-value*** |
| Intercept | **-0.03 (*0.01*)** | **-3.42 (2940.67)** | **.001** |
| Same-day stress impact | **0.13 (*0.02*)** | **8.21 (359.49)** | **<.001** |
| Stress impact from two days ago | 0.03 (*0.01*) | 2.38 (315.26) | .02 |
| Major life stress over 49 days | <0.01 (*0.01*) | 0.03 (2932.01) | .98 |
| Same-day stress impact X major life stress  over 49 days | <0.01 (*0.01*) | -0.13 (349.06) | .90 |
| Stress impact from two days ago X major  life stress over 49 days | 0.01 (*0.01*) | 0.55 (332.59) | .58 |
| Negative affect | **0.13 (*0.02*)** | **8.50 (417.74)** | **<.001** |
| BMI | 0.01 (*0.01*) | 0.74 (4223.28) | .46 |
| Intercept | **-0.03 (*0.01*)** | **-3.41 (2930.84)** | **.001** |
| Same-day stress impact | **0.13 (*0.02*)** | **8.23 (354.24)** | **<.001** |
| Stress impact from two days ago | 0.03 (*0.01*) | 2.43 (308.35) | .02 |
| Major life stress across the lifetime | <0.01 (*0.01*) | 0.05 (2955.46) | .96 |
| Same-day stress impact X major life stress  across the lifetime | <0.01 (*0.02*) | 0.15 (344.43) | .88 |
| Stress impact from two days ago X major  life stress across the lifetime | 0.01 (*0.01*) | 0.97 (312.38) | .33 |
| Negative affect | **0.13 (*0.02*)** | **8.50 (417.67)** | **<.001** |
| BMI | 0.01 (*0.01*) | 0.72 (4225.50) | .47 |

*Note*: MLM = multilevel model; BMI = body mass index; stress impact = daily stress impact.

Values significant at *p* <.01 are bolded.

**Table S4.** *Between-person Pearson correlations for average daily stress, major life stress, emotional eating, and covariates (N = 477).*

|  | 1. | 2. | 3. | 4. | 5. | 6. | 7. |
| --- | --- | --- | --- | --- | --- | --- | --- |
| **Daily Variables** |  |  |  |  |  |  |  |
| 1. Avg. emotional eating | 1.00 | **0.56***** | **0.54***** | 0.07 | 0.05 | -0.10* | 0.10 |
| 1. Avg. stress impact | -- | 1.00 | **0.67***** | **0.33***** | 0.03 | -0.12* | -0.02 |
| 1. Avg. negative affect | -- | -- | 1.00 | **0.28***** | -0.02 | **-0.24***** | -0.04 |
|  |  |  |  |  |  |  |  |
| **Non-Daily Variables** |  |  |  |  |  |  |  |
| 1. Avg. Major life stress in last 12 months | -- | -- | -- | 1.00 | 0.09 | -0.05 | **-0.18***** |
| 1. Avg. BMI | -- | -- | -- | -- | 1.00 | **0.31***** | -0.09 |
| 1. Age | -- | -- | -- | -- | -- | 1.00 | -0.10* |
| 1. Income | -- | -- | -- | -- | -- | -- | 1.00 |

*Note:* Avg. = average; BMI = body mass index averaged across the three measurements at the beginning, middle, and end of the 49-day collection period; stress impact = daily stress impact. All daily variables were averaged across the 49 days of the study. **p* <.05; ****p* <.01

**Table S5.** *Within-person Pearson correlations for same-day and time-lagged daily stress impact, daily emotional eating, and covariates (N = 477).*

| **Variables** | 1. | 2. | 3. | 4. | 5. |
| --- | --- | --- | --- | --- | --- |
| **Same-day daily variables** |  |  |  |  |  |
| 1. Emotional eating | 1.00 | **0.22***** | **0.18***** | **0.06***** | **0.05***** |
| 1. Same-day stress impact | -- | 1.00 | **0.46***** | **0.13***** | **0.10***** |
| 1. Negative affect | -- | -- | 1.00 | **0.07***** | **0.05***** |
| **Time-lagged daily stress variables** |  |  |  |  |  |
| 1. Stress impact 1 day ago | -- | -- | -- | 1.00 | **0.79***** |
| 1. Stress impact 2 days ago | -- | -- | -- | -- | 1.00 |

*Note:* ****p* <.01.**Table S6.** *Pearson correlations for hair cortisol concentration (HCC), emotional eating, and covariates in the full sample of HCC participants (N = 234) and subsample of HCC participants without confounding factors for HCC (N = 220).*

| **Full sample (N = 234)** | 1. | 2. | 3. | 4. | 5. | 6. | 7. | 8. | 9. |
| --- | --- | --- | --- | --- | --- | --- | --- | --- | --- |
| 1. HCC | 1.00 | -0.13 | 0.02 | 0.07 | 0.06 | -0.05 | 0.05 | <0.01 | 0.09 |
| 1. Avg. emotional eating | -- | 1.00 | **0.58***** | 0.11 | **0.62***** | 0.10 | -0.12 | 0.03 | <0.01 |
| 1. Avg. stress impact | -- | -- | 1.00 | **0.32***** | **0.75***** | 0.13* | -0.14* | -0.07 | -0.01 |
| 1. Major life stress in the last 12 months | -- | -- | -- | 1.00 | **0.22***** | 0.16* | 0.13* | <0.01 | 0.09 |
| 1. Avg. negative affect | -- | -- | -- | -- | 1.00 | 0.08 | **-0.24***** | <0.01 | 0.01 |
| 1. Avg. BMI | -- | -- | -- | -- | -- | 1.00 | **0.28***** | -0.09 | -0.12 |
| 1. Age | -- | -- | -- | -- | -- | -- | 1.00 | -0.14* | **-0.26***** |
| 1. Avg. hours of sleep/night | -- | -- | -- | -- | -- | -- | -- | 1.00 | 0.11 |
| 1. Income | -- | -- | -- | -- | -- | -- | -- | -- | 1.00 |
| **Subsample of women without confounding factors for HCC (N = 220)** | 1. | 2. | 3. | 4. | 5. | 6. | 7. | 8. | 9. |
| 1. HCC | 1.00 | -0.15* | 0.01 | 0.03 | 0.06 | -0.06 | 0.04 | <0.01 | 0.10 |
| 1. Avg. emotional eating | -- | 1.00 | **0.58***** | 0.02 | **0.62***** | 0.08 | -0.15* | 0.03 | 0.01 |
| 1. Avg. stress impact | -- | -- | 1.00 | **0.26***** | **0.76***** | 0.13 | -0.15* | -0.08 | -0.01 |
| 1. Major life stress in the last 12 months | -- | -- | -- | 1.00 | 0.10 | 0.12 | 0.05 | 0.13 | 0.05 |
| 1. Avg. negative affect | -- | -- | -- | -- | 1.00 | 0.08 | **-0.26***** | <0.01 | 0.01 |
| 1. Avg. BMI | -- | -- | -- | -- | -- | 1.00 | **0.27***** | -0.09 | -0.11 |
| 1. Age | -- | -- | -- | -- | -- | -- | 1.00 | -0.13 | **-0.24***** |
| 1. Avg. hours of sleep/night | -- | -- | -- | -- | -- | -- | -- | 1.00 | -0.13 |
| 1. Income | -- | -- | -- | -- | -- | -- | -- | -- | 1.00 |

*Note:* Avg. = average; BMI = body mass index averaged across the three measurements at the beginning, middle, and end of the 49-day collection period; HCC = hair cortisol concentration.

All daily variables were averaged across the 49 days of the study. **p* <.05; ****p* <.01

**Table S7.** *Results from the post-hoc within-person MLMs examining predictive effects of emotional eating from one and two days ago on subsequent stress impact (N = 477).*

| **Emotional Eating from One Day Ago** | | | |
| --- | --- | --- | --- |
| **Predictors** | **β (*SD*)** | **t (df)** | ***p-value*** |
| Intercept | -0.02 (*0.01*) | -2.58 (2987.48) | .01 |
| Same-day emotional eating | **0.09 (*0.01*)** | **8.73 (288.74)** | **<.001** |
| Emotional eating from one day ago | **0.03 (*0.01*)** | **3.92 (263.40)** | **<.001** |
| Avg. negative affect | **0.42 (*0.01*)** | **35.22 (292.04)** | **<.001** |
| Avg. BMI | 0.01 (*0.01*) | 0.83 (273.23) | .41 |
| **Emotional Eating from Two Days Ago** | | | |
| **Predictors** | **β (*SD*)** | **t (df)** | ***p-value*** |
| Intercept | **-0.03 (*0.01*)** | **-4.08 (2861.87)** | **<.001** |
| Same-day emotional eating | **0.09 (*0.01*)** | **7.96 (284.70)** | **<.001** |
| Emotional eating from two days ago | 0.02 (*0.01*) | 2.39 (261.92) | .02 |
| Avg. negative affect | **0.42 (*0.01*)** | **33.82 (296.87)** | **<.001** |
| Avg. BMI | 0.02 (*0.01*) | 1.57 (272.89) | .12 |

*Note:* Avg. = average; BMI = body mass index. All daily variables were averaged across the 49 days of the study. Values significant at *p* <.01 are bolded.

**Table S8.** *Results from between-person MLMs examining main and interactive effects of stress on average levels of emotional eating, including lifetime ED diagnosis as a covariate (N = 419).*

| **Main Effects** | | | |
| --- | --- | --- | --- |
| **Variables** | **β (*SD*)** | **t (df)** | ***p-value*** |

| Intercept | **-0.13 (*0.05*)** | **-2.97 (199.95)** | **.003** |
| --- | --- | --- | --- |
| Avg. stress impact | **0.36 (*0.06*)** | **5.63 (156.85)** | **<.001** |
| Avg. negative affect | **0.21 (*0.07*)** | **3.24 (201.00)** | **.001** |
| Avg. BMI | 0.02 (*0.04*) | 0.53 (288.88) | .60 |
| Age | <0.01 (*0.05*) | -0.04 (210.66) | .97 |
| Income | 0.04 (*0.05*) | 0.89 (180.88) | .37 |
| Lifetime DSM-5 ED | **0.49 *(0.15)*** | **3.25 (313.79)** | **.001** |
| Intercept | -0.12 (*0.05*) | -2.46 (206.45) | .02 |
| Major life stress in the last 12 months | -0.02 (*0.05*) | -0.32 (98.85) | .75 |
| Avg. negative affect | **0.47 (*0.06*)** | **8.35 (160.85)** | **<.001** |
| Avg. BMI | 0.06 (*0.05*) | 1.30 (264.72) | .20 |
| Age | -0.03 (*0.05*) | -0.49 (208.42) | .62 |
| Income | 0.05 (*0.05*) | 1.07 (182.78) | .29 |
| Lifetime DSM-5 ED | 0.39 *(0.16)* | 2.45 (276.22) | .02 |

| **Interactions** | | | | |
| --- | --- | --- | --- | --- |
|  | **β (*SD*)** | **t (df)** | | ***p-value*** |
| Intercept | **-0.17 (*0.05*)** | | **-3.74 (207.98)** | **<.001** |
| Avg. stress impact | **0.39 (*0.07*)** | | **5.96 (160.55)** | **<.001** |
| Major life stress in the last 12 months | -0.09 (*0.05*) | | -1.87 (62.43) | .07 |
| Avg. stress impact X major life stress  in the last 12 months | 0.10 (*0.05*) | | 1.90 (77.37) | .06 |
| Avg. negative affect | **0.21 (*0.07*)** | | **3.25 (196.60)** | **.001** |
| Avg. BMI | 0.04 (*0.04*) | | 0.89 (263.02) | .37 |
| Age | <0.01 *(0.05)* | | -0.25 (207.55) | .80 |
| Income | 0.04 (*0.05*) | | 0.79 (182.22) | .43 |
| Lifetime DSM-5 ED | **0.44 *(0.15)*** | | **2.89 (276.87)** | **.004** |

*Note:* MLM = multilevel model; avg. = average; BMI = body mass index; stress impact = daily stress impact; ED = anorexia nervosa, bulimia nervosa, or binge-eating disorder (*n* = 32 with lifetime ED). All daily variables were averaged across the 49 days of data collection, and BMI was averaged across the 3 study assessments. The N is lower than the total study N because some participants had missing data on the SCID diagnostic interview used to assess eating disorder diagnoses. Participants with a lifetime ED (*p* = .024, *d* = .51) reported higher mean emotional eating than women without lifetime EDs. Betas represent standardized effects. Effects significant at *p* <.01 are bolded.

**Table S9.** *Results from between-person MLMs examining main and interactive effects of stress on average levels of emotional eating, including presence of BE as a covariate (N = 452).*

| **Main Effects** | | | |
| --- | --- | --- | --- |
| **Variables** | **β (*SD*)** | **t (df)** | ***p-value*** |

| Intercept | -0.12 (*0.05*) | -2.61 (204.52) | .010 |
| --- | --- | --- | --- |
| Avg. stress impact | **0.31 (*0.06*)** | **5.31 (140.30)** | **<.001** |
| Avg. negative affect | **0.26 (*0.07*)** | **4.01 (212.06)** | **<.001** |
| Avg. BMI | <0.01 (*0.04*) | -0.03 (307.52) | .98 |
| Age | -0.02 (*0.05*) | -0.48 (201.17) | .63 |
| Income | 0.02 (*0.04*) | 0.47 (183.78) | .64 |
| Lifetime BE | **0.50 *(0.12)*** | **4.05 (342.90)** | **<.001** |
| Intercept | -0.11 (*0.05*) | -2.37 (209.90) | .02 |
| Major life stress in the last 12 months | <0.01 (*0.05*) | -0.20 (67.97) | .84 |
| Avg. negative affect | **0.49 (*0.06*)** | **8.68 (162.75)** | **<.001** |
| Avg. BMI | 0.02 (*0.04*) | 0.49 (285.90) | .63 |
| Age | -0.04 (*0.05*) | -0.73 (204.00) | .47 |
| Income | 0.02 (*0.05*) | 0.43 (180.36) | .67 |
| Lifetime BE | **0.53 *(0.13)*** | **4.12 (328.16)** | **<.001** |

| **Interactions** | | | | |
| --- | --- | --- | --- | --- |
|  | **β (*SD*)** | **t (df)** | | ***p-value*** |
| Intercept | -0.16 (*0.05*) | | -3.43 (212.31) | **<.001** |
| Avg. stress impact | **0.36 (*0.06*)** | | **5.80 (147.72)** | **<.001** |
| Major life stress in the last 12 months | -0.09 (*0.05*) | | -2.01 (332.18) | .05 |
| Avg. stress impact X major life stress  in the last 12 months | 0.08 (*0.05*) | | 1.72 (104.92) | .09 |
| Avg. negative affect | **0.25 (*0.06*)** | | **3.87 (209.31)** | **<.001** |
| Avg. BMI | 0.01 (*0.04*) | | 0.25 (293.44) | .81 |
| Age | -0.02 *(0.05)* | | -0.48 (199.30) | .63 |
| Income | 0.01 (*0.04)* | | 0.32 (190.37) | .75 |
| Lifetime BE | **0.51 *(0.12)*** | | **4.18 (332.90)** | **<.001** |

*Note:* MLM = multilevel model; avg. = average; BMI = body mass index; stress impact = daily stress impact; lifetime BE = lifetime history of objective binge eating episodes (*n* = 45 with lifetime BE). All daily variables were averaged across the 49 days of data collection, and BMI was averaged across the 3 study assessments. The N is lower than the total study N because some participants had missing data on the SCID diagnostic interview used to assess presence of BE. Participants with lifetime BE (*p* <.001; *d* = .68) reported higher mean emotional eating than women without lifetime BE. Betas represent standardized effects. Effects significant at *p* <.01 are bolded.

**Table S10.** *Results from the within-person MLMs examining main and interactive effects of the same-day and time-lagged stress variables and covariates on daily levels of emotional eating in the subsample of participants with lifetime EDs (N = 32).*

| **Same-Day Daily Stress Impact** | | | |
| --- | --- | --- | --- |
| **Main Effects** | | | |
| **Variables** | **β (*SD*)** | **t (df)** | ***p-value*** |

| Intercept | <0.01 (*0.04*) | -0.09 (142.16) | .93 |
| --- | --- | --- | --- |
| Same-day stress impact | 0.09 (*0.06*) | 1.63 (14.93) | .12 |
| Negative affect | 0.14 (*0.07*) | 2.16 (17.32) | .05 |
| BMI | 0.03 (*0.08*) | 0.34 (12.30) | .74 |

| **Interactions** | | | |
| --- | --- | --- | --- |
|  | **β (*SD*)** | **t (df)** | ***p-value*** |
| Intercept | <0.01 (*0.04*) | 0.05 (144.00) | .96 |
| Same-day stress impact | 0.10 (*0.06*) | 1.75 (15.56) | .10 |
| Major life stress in the last 12 months | -0.01 (*0.06*) | -0.21 (140.05) | .84 |
| Same-day stress impact X major life stress  in the last 12 months | 0.07 (*0.09*) | .77 (15.89) | .45 |
| Negative affect | 0.14 (*0.07*) | 2.16 (17.34) | .05 |
| BMI | 0.03 (*0.08*) | 0.35 (12.29) | .73 |
| **Daily Stress from One Day Ago (Lagged Effects)** | | | |
| **Main Effects** | | | |
|  | **β (*SD*)** | **t (df)** | ***p-value*** |
| Intercept | -0.04 (*0.04*) | -0.91 (143.87) | .37 |
| Same-day stress impact | 0.07 (*0.06*) | 1.24 (14.27) | .24 |
| Stress impact from one day ago | -0.02 (*0.04*) | -0.60 (231.92) | .55 |
| Negative affect | 0.14 (*0.07*) | 2.21 (16.89) | .04 |
| BMI | 0.05 (*0.06*) | 0.76 (11.78) | .47 |
| **Interactions** | | | |
|  | **β (*SD*)** | **t (df)** | ***p-value*** |
| Intercept | -0.02 (*0.05*) | -.35 (181.49) | .73 |
| Same-day stress impact | 0.10 (*0.06*) | 1.71 (16.38) | .11 |
| Stress impact from one day ago | -0.06 (*0.06*) | -1.00 (311.93) | .32 |
| Major life stress in the last 12 months | 0.07 (*0.08*) | 0.80 (182.78) | .42 |
| Same-day stress impact X major life stress  in the last 12 months | 0.14 (*0.09*) | 1.60 (16.45) | .13 |
| Stress impact from one day ago X major life  stress in the last 12 months | 0.01 (*0.07*) | .15 (252.09) | .88 |
| Negative affect | 0.14 (*0.06*) | 2.20 (16.93) | .04 |
| BMI | 0.05 (*0.06*) | 0.74 (11.36) | .47 |
| **Daily Stress from Two Days Ago (Lagged Effects)** | | | |
| **Main Effects** | | | |
|  | **β (*SD*)** | **t (df)** | ***p-value*** |
| Intercept | -0.04 (*0.04*) | -1.04 (145.48) | .30 |
| Same-day stress impact | 0.07 (*0.06*) | 1.18 (13.50) | .26 |
| Stress impact from two days ago | 0.04 (*0.04*) | 1.03 (234.95) | .30 |
| Negative affect | 0.12 (*0.07*) | 1.82 (16.61) | .09 |
| BMI | 0.03 (*0.06*) | 0.47 (13.28) | .64 |
| **Interactions** | | | |
|  | **β (*SD*)** | **t (df)** | ***p-value*** |
| Intercept | -0.02 (*0.05*) | -.34 (181.05) | .73 |
| Same-day stress impact | 0.09 (*0.06*) | 1.46 (14.77) | .17 |
| Stress impact from two days ago | 0.03 (*0.06*) | 0.60 (308.01) | .55 |
| Major life stress in the last 12 months | 0.06 (*0.08*) | 0.69 (181.73) | .49 |
| Same-day stress impact X major life stress  in the last 12 months | 0.11 (*0.09*) | 1.19 (14.82) | .26 |
| Stress impact from two days ago X major  life stress in the last 12 months | -0.03 (*0.07*) | -0.51 (252.86) | .61 |
| Negative affect | 0.12 (*0.07*) | 1.78 (16.51) | .09 |
| BMI | 0.03 (*0.06*) | 0.48 (12.97) | .64 |

*Note*: MLM = multilevel model; BMI = body mass index; stress impact = daily stress impact; ED = anorexia nervosa, bulimia nervosa, or binge-eating disorder. Betas represent standardized effects. Because all participants in this subsample had lifetime EDs, ED status was not included as a covariate in the models. Note that associations may not be significant even when of similar magnitude to those observed in the full sample due to smaller sample size. Effects significant at *p* <.01 are bolded.

**Table S11.** *Results from the within-person MLMs examining main and interactive effects of the same-day and time-lagged stress variables and covariates on daily levels of emotional eating in the subsample of participants with lifetime BE (N = 45).*

| **Same-Day Daily Stress Impact** | | | |
| --- | --- | --- | --- |
| **Main Effects** | | | |
| **Variables** | **β (*SD*)** | **t (df)** | ***p-value*** |

| Intercept | <0.01 (*0.03*) | -0.09 (252.61) | .93 |
| --- | --- | --- | --- |
| Same-day stress impact | **0.12 (*0.04*)** | **3.32 (28.15)** | **.002** |
| Negative affect | 0.12 (*0.05*) | 2.63 (34.89) | .01 |
| BMI | <0.01 (*0.04*) | 0.13 (22.33) | .90 |

| **Interactions** | | | |
| --- | --- | --- | --- |
|  | **β (*SD*)** | **t (df)** | ***p-value*** |
| Intercept | <0.01 (*0.03*) | -0.09 (249.42) | .93 |
| Same-day stress impact | **0.12 (*0.04*)** | **3.36 (26.93)** | **.002** |
| Major life stress in the last 12 months | 0.01 (*0.05*) | 0.25 (246.20) | .80 |
| Same-day stress impact X major life stress  in the last 12 months | 0.05 (*0.05*) | 1.07 (25.48) | .30 |
| Negative affect | 0.12 (*0.04*) | 2.60 (34.95) | .01 |
| BMI | <0.01 (*0.05*) | 0.17 (22.30) | .87 |
| **Daily Stress from One Day Ago (Lagged Effects)** | | | |
| **Main Effects** | | | |
|  | **β (*SD*)** | **t (df)** | ***p-value*** |
| Intercept | -0.06 (*0.03*) | -1.99 (271.69) | .05 |
| Same-day stress impact | 0.09 (*0.03*) | 2.77 (24.21) | .01 |
| Stress impact from one day ago | 0.04 (*0.03*) | 1.21 (486.87) | .23 |
| Negative affect | **0.13 (*0.04*)** | **2.99 (33.61)** | **.005** |
| BMI | <0.01 (*0.04*) | 0.05 (18.44) | .97 |
| **Interactions** | | | |
|  | **β (*SD*)** | **t (df)** | ***p-value*** |
| Intercept | -0.06 (*0.03*) | -1.84 (281.09) | .07 |
| Same-day stress impact | 0.09 (*0.03*) | 2.77 (23.89) | .01 |
| Stress impact from one day ago | 0.05 (*0.04*) | 1.20 (551.66) | .23 |
| Major life stress in the last 12 months | <0.01 (*0.05*) | -0.07 (288.13) | .95 |
| Same-day stress impact X major life stress  in the last 12 months | 0.03 (*0.05*) | 0.71 (22.27) | .49 |
| Stress impact from one day ago X major life  stress in the last 12 months | -0.02 (*0.05*) | -0.32 (480.41) | .75 |
| Negative affect | **0.13 (*0.04*)** | **2.96 (33.55)** | **.006** |
| BMI | <0.01 (*0.04*) | 0.08 (17.81) | .94 |
| **Daily Stress from Two Days Ago (Lagged Effects)** | | | |
| **Main Effects** | | | |
|  | **β (*SD*)** | **t (df)** | ***p-value*** |
| Intercept | -0.07 (*0.03*) | -2.42 (269.41) | .02 |
| Same-day stress impact | **0.10 (*0.03*)** | **2.84 (26.00)** | **.009** |
| Stress impact from two days ago | 0.07 (*0.03*) | 2.31 (482.78) | .02 |
| Negative affect | 0.09 (*0.04*) | 2.12 (32.40) | .04 |
| BMI | <0.01 (*0.04*) | 0.09 (21.05) | .93 |
| **Interactions** | | | |
|  | **β (*SD*)** | **t (df)** | ***p-value*** |
| Intercept | -0.07 (*0.03*) | -2.13 (275.72) | .03 |
| Same-day stress impact | 0.10 (*0.03*) | 2.78 (25.98) | .01 |
| Stress impact from two days ago | **0.10 (*0.04*)** | **2.72 (541.97)** | **.007** |
| Major life stress in the last 12 months | -0.02 (*0.05*) | -0.36 (279.94) | .72 |
| Same-day stress impact X major life stress  in the last 12 months | 0.03 (*0.05*) | 0.63 (24.76) | .54 |
| Stress impact from two days ago X major  life stress in the last 12 months | -0.06 (*0.05*) | -1.22 (475.36) | .22 |
| Negative affect | 0.08 (*0.04*) | 2.07 (32.41) | .05 |
| BMI | <0.01 (*0.04*) | 0.06 (20.26) | .95 |

*Note*: MLM = multilevel model; BMI = body mass index; stress impact = daily stress impact; lifetime BE = lifetime history of objective binge eating episodes. Betas represent standardized effects. Because all participants in this subsample had lifetime BEs, BE status was not included as a covariate in the models. Note that associations may not be significant even when of similar magnitude to those observed in the full sample due to smaller sample size. Effects significant at *p* <.01 are bolded.
